# Effects of Suramin on Polycystic Kidney Disease in a Mouse Model of Polycystin-1 Deficiency

**DOI:** 10.3390/ijms23158499

**Published:** 2022-07-31

**Authors:** Ming-Yang Chang, Shen-Hsing Hsu, Li-Yi Ma, Li-Feng Chou, Cheng-Chieh Hung, Ya-Chung Tian, Chih-Wei Yang

**Affiliations:** Kidney Research Center, Department of Nephrology, Chang Gung Memorial Hospital, Chang Gung University College of Medicine, Taoyuan 333, Taiwan; d938208@gmail.com (S.-H.H.); maliyi24@gmail.com (L.-Y.M.); d928208@gmail.com (L.-F.C.); cchung@adm.cgmh.org.tw (C.-C.H.); dryctian@adm.cgmh.org.tw (Y.-C.T.); cwyang@ms1.hinet.net (C.-W.Y.)

**Keywords:** polycystic kidney disease, purinergic signaling, suramin

## Abstract

The aberrant activation of the purinergic signaling pathway has been shown to promote cyst growth and fluid secretion in autosomal dominant polycystic kidney disease (ADPKD). Suramin is an anti-parasitic drug that has strong anti-purinergic properties. Whether suramin could have a therapeutic effect on ADPKD has not been fully investigated. We examined the effect of suramin on cyst progression in a *Pkd1* microRNAs transgenic mouse model that presented stable *Pkd1* knockdown and moderate disease progression. The *Pkd1*-deficient mice were treated with suramin (60 mg/kg) by intraperitoneal injection twice a week from postnatal days 35 to 90. Kidney-to-body weight ratios, cyst indices, and blood urea nitrogen (BUN) levels were measured. Cell proliferation and macrophage infiltration were determined by immunohistochemistry. The suramin-treated group had significantly lower renal cyst densities, cell proliferation, and macrophage infiltration compared with saline-treated controls. Suramin significantly inhibited ERK phosphorylation and the expression of *Il1b*, *Il6*, *Nlrp3*, *Tgfb*, *Fn1*, *P2rx7*, and *P2ry2* mRNAs in the kidneys. However, BUN levels remained high despite the reduction in cyst growth. Furthermore, plasma cystatin C and neutrophil gelatinase-associated lipocalin (NGAL) levels were significantly higher in the suramin-treated group compared with the control group. Periodic acid-Schiff staining revealed degenerative changes and epithelial cell vacuolation in the non-cystic renal tubules, which indicated phospholipidosis following suramin treatment. These results suggest that suramin may reduce renal cyst growth and inflammation, but the associated tubular cell injuries could limit its therapeutic potential. Other purinergic receptor antagonists with less nephrotoxicity may deserve further investigation for the treatment of ADPKD.

## 1. Introduction

Autosomal dominant polycystic kidney disease (ADPKD) is a common genetic disease characterized by the formation of multiple renal cysts and the progressive loss of kidney function, leading to end-stage kidney failure in about 50% of affected patients over the age of 60 [1,2]. The majority of ADPKD patients (~85%) are caused by mutations in *PKD1* and the remaining are caused by *PKD2* and other novel genes, such as *GANAB* and *DNAJB11* [3,4]. The pathogenesis of ADPKD involves excessive cell proliferation and the fluid secretion of the cyst-lining epithelium, which has been associated with the aberrant activation of cyclic adenosine monophosphate (cAMP), mTOR (mammalian target of rapamycin), and other pro-proliferative signaling pathways [5,6]. Tolvaptan, a vasopressin V2 receptor antagonist, is the first FDA-approved treatment for ADPKD [7]. However, the potential liver toxicity and severe aquaretic side effects have limited its clinical use in ADPKD patients with rapid disease progression [8]. Therefore, other potential treatments targeting different signaling pathways warrant further investigation [9].

The purinergic P2 receptor signaling pathway has been found to enhance fluid secretion and cell proliferation in ADPKD [10,11]. High levels of adenosine-5′-triphosphate (ATP) in renal cysts reduce the sodium reabsorption mediated by epithelial sodium channels (ENaC) and promote cyst expansion [12]. A synergistic effect of cAMP and ATP on enhancing fluid secretion and cyst growth has also been found in the Madin-Darby canine kidney (MDCK) cell model [13]. An in vivo study showed that P2X7 receptor knockout attenuates cyst growth in a rat model of autosomal recessive PKD (ARPKD) [14]. P2X7 receptor inhibition also reduces cell proliferation and pronephros cyst formation in a *Pkd2*-deficient zebrafish model [15]. Furthermore, the P2Y2 receptors are transcriptionally regulated by hypoxia-inducible factor (HIF)-1α and promote ATP-dependent cyst growth [16]. These data suggest a potential benefit of blocking purinergic P2 receptors in slowing cyst growth in ADPKD.

Suramin is a 100-year-old drug that has been primarily used for the treatment of African trypanosomiasis [17,18]. It is a non-selective P2 receptor antagonist (P2Xs and P2Ys) that has multitarget and pleiotropic effects [19,20,21,22]. Suramin was initially investigated as an antitumor and anti-HIV (human immunodeficiency virus) agent but has demonstrated limited efficacy in previous clinical trials [23,24]. Suramin has been repurposed to improve the metabolic abnormalities in autism spectrum disorder (ASD) via the inhibition of P2X7 receptors [25]. Furthermore, single-dose suramin reduced the deposition of extracellular matrix and interstitial leukocyte infiltration in a mouse model of unilateral ureteral obstruction (UUO), suggesting its therapeutic potential for chronic kidney diseases [26]. An in vitro study showed that suramin reduces cyst formation in a three-dimensional microcyst model in MDCK cells [13]. Therefore, we hypothesized that suramin might suppress ADPKD by inhibiting purinergic receptor pathways in vivo.

In this study, we determined the effects of suramin on cyst growth and disease progression in an established *Pkd1* hypomorphic model of ADPKD. We examined if suramin treatment could ameliorate the cystic burden of *Pkd1* knockdown mice and suppress renal inflammation and cell proliferation. We also evaluated the degree of renal tubular injury and the overall kidney function following suramin treatment.

## 2. Results

### 2.1. Increased Expression of P2X7 Receptors in Pkd1-miR Tg Mice

**Figure 1:**
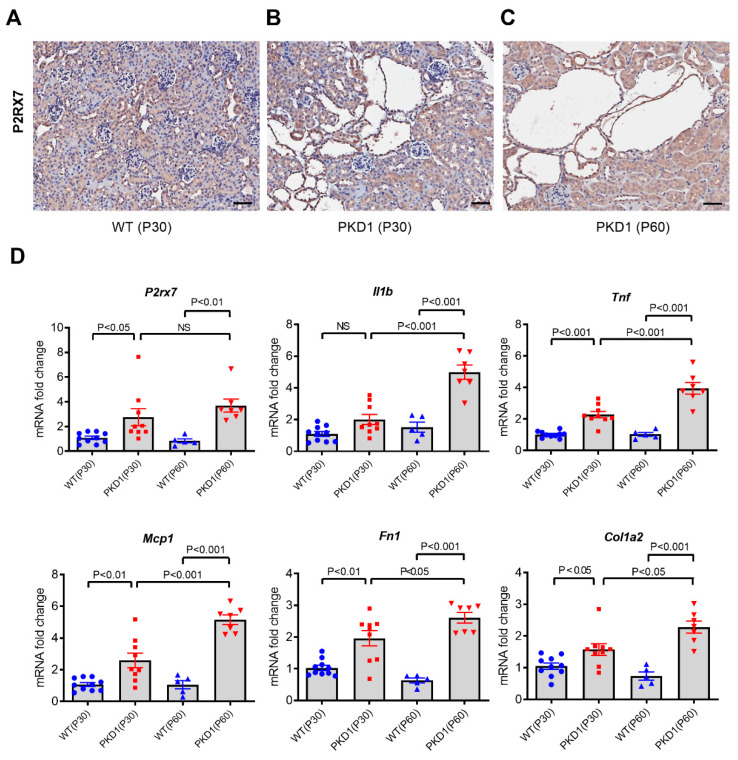
The expression of P2X7 receptors in wild-type and *Pkd1-*miR Tg mice. (**A**) Representative images of kidney sections showing diffuse immunostaining for P2X7 receptors in renal tubules in wild-type mice (n = 5). (**B**) Strong staining for P2X7 receptors is noted in the cyst-lining epithelial cells in the *Pkd1* miR Tg mice at 30 days of postnatal age (n = 5). (**C**) Diffuse staining for P2X7 receptors in cyst walls and non-cystic tubules in the *Pkd1*-miR Tg mice at 60 days of age (n = 5). (**D**) Comparison of the renal mRNA expression of *P2rx7* and other pro-inflammatory and pro-fibrotic genes in the kidneys of wild-type and *Pkd1* miR Tg mice according to different postnatal ages (n = 10, 9, 5, 7, respectively). Data are mean ± SEM. Scale bar, 50 µm.

This study investigated the effects of suramin on cystogenesis in an established mouse model of *Pkd1* knockdown that recapitulates the chronic disease progression in humans [27]. First, we examined whether the expression of P2X7 receptors is upregulated in *Pkd1*-miR Tg mice as has been previously shown in the *Pkd1*^RC/RC^ mouse model [12]. In wild-type mice, we found a strong expression of P2X7 receptors in the distal tubules and a weaker expression in the proximal tubules by immunohistochemistry, whereas the signal was undetectable in the glomeruli (Figure 1A). In *Pkd1*-miR Tg mice, the immunostaining of P2X7 receptors was most evident in the cyst lining epithelium both at the ages of postnatal day 30 (P30) and P60 (Figure 1B,C). Furthermore, *Pkd1*-miR Tg mice had significantly higher levels of renal *P2rx7* mRNA expression than wild-type mice by 2.5-fold at P30 (*p* < 0.05) and 4.4-fold at P60 (*p* < 0.01), respectively. This was associated with an increased mRNA expression of *Il1b*, *Tnf*, *Mcp1*, *Fn1*, and *Col1a2* genes in the cystic kidneys of *Pkd1*-miR Tg mice (Figure 1D).

### 2.2. Suramin Suppressed Cystogenesis in Pkd1-miR Tg Mice

**Figure 2:**
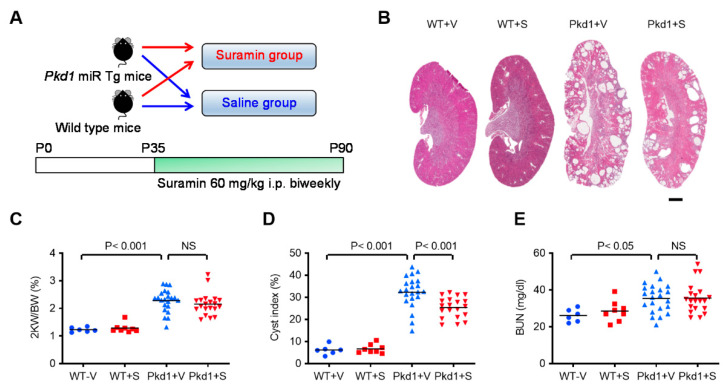
**Suramin treatment reduces cyst burden in *Pkd1* miR Tg mice.** (**A**) Schematic illustration of the experimental groups and the time frame of suramin administration. (**B**) Representative images of whole kidney sections (H&E stain) in the suramin-treated *Pkd1*-miR Tg mice compared to the vehicle-treated controls. (**C**) Kidney-to-body weight ratios, (**D**) cystic indices, and (**E**) BUN levels in different experimental groups as indicated (n = 6, 8, 22, and 20 per group, respectively). Data are mean ± SEM. S, suramin; V, vehicle. Scale bar, 1 mm.

We then investigated the effects of suramin on cyst formation in *Pkd1-*miR Tg mice (Figure 2A). The dosage of suramin (60 mg/kg twice weekly i.p.) was selected according to previous studies that effectively reduce myocardial fibrosis, inflammation, and myonecrosis in the mdx mouse model of Duchenne muscular dystrophy [28,29,30]. As shown in Figure 2B and Supplementary Table S1, the suramin-treated *Pkd1*-miR Tg mice tended to have reduced kidney weights compared with saline-treated mutant mice, but the kidney-to-body weight ratios were not significantly different between the control and treatment groups (Figure 2C), possibly due to lower body weights found in the suramin-treated mice. Suramin treatment could cause systemic adverse effects that may affect the normal growth and metabolism in mice [31]. We also performed a histomorphological analysis on whole kidney sections for the comparison of cyst indices among groups. Indeed, *Pkd1-*miR Tg mice treated with suramin had significantly reduced cyst indices (21.4 % reduction) compared with vehicle-treated mutant mice (*p* < 0.001) (Figure 2D). However, the elevated BUN levels in *Pkd1-*miR Tg mice were not ameliorated after treatment with suramin (Figure 2E). These results indicated that suramin suppressed renal cyst formation in *Pkd1*-miR Tg mice but did not slow the decline of kidney function.

### 2.3. Effect of Suramin on Cell Proliferation

**Figure 3:**
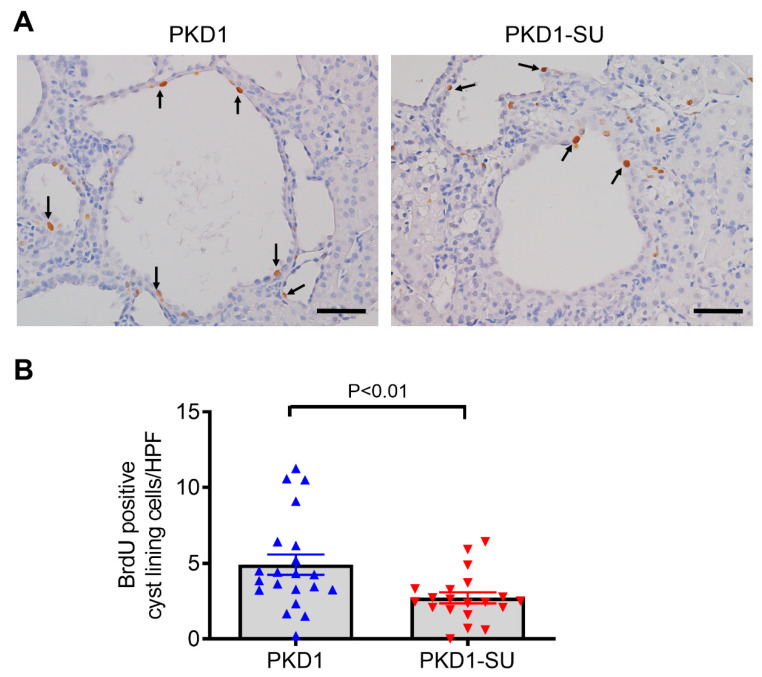
Suramin treatment inhibits the proliferation of cyst-lining epithelial cells in *Pkd1* miR Tg mice. (**A**) Immunostaining for BrdU incorporation in kidney sections from *Pkd1*-miR Tg mice in vehicle- and suramin-treated groups. Arrows indicate BrdU-positive cyst lining epithelial cells. Scale bar, 50 µm. (**B**) Quantitative image analysis showed a significant reduction (*p* < 0.01) in the number of BrdU-positive cells in the suramin-treated *Pkd1*-miR Tg mice (n = 20) compared with the vehicle-treated mutant mice (n = 21) using an unpaired, two-tailed *t*-test. Data are mean ± SEM.

Because suramin has been shown to suppress tumor growth in earlier research [32], we next sought to determine whether suramin could reduce the proliferation of cyst-lining epithelial cells. We used in vivo BrdU labeling assays to quantify cells in the S-phase of cell cycle progression. A significant reduction (44.7%) in the number of BrdU-labeled cyst-lining cells was detected in *Pkd1*-miR Tg mice with suramin treatment compared with the saline-treated group (*p < 0.01*) (Figure 3). This result suggests that suramin could reduce cyst densities by inhibiting cell proliferation in the cyst-lining epithelium.

### 2.4. Suramin Inhibited Renal Inflammation and Macrophage Infiltration

**Figure 4:**
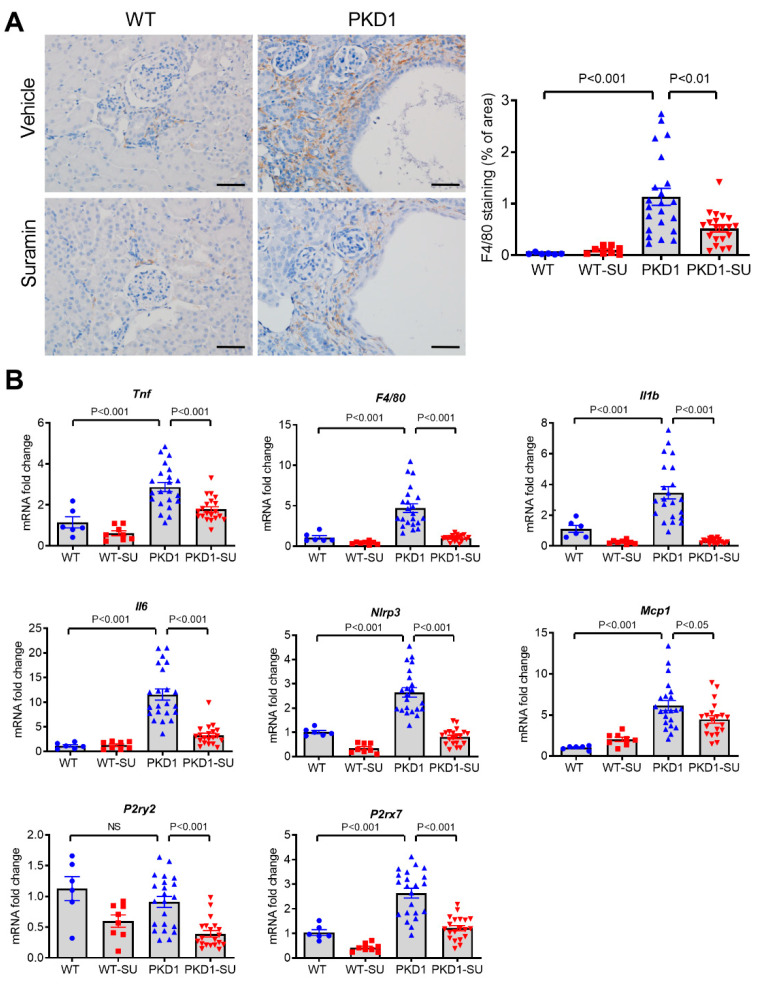
Suramin treatment inhibited renal inflammation and macrophage infiltration. (**A**) Immunostaining of kidney sections for F4/80 in different treatment groups as indicated. Quantitative image analysis showed a significant decrease in the percentage of F4/80 positive cells in the suramin-treated *Pkd1*-miR Tg mice compared with the vehicle-treated controls. Scale bar, 50 µm. (**B**) Suramin treatment reduced the mRNA expression of pro-inflammatory genes and purinergic receptor genes in the kidneys, as determined by qRT-PCR (n = 6, 8, 22, and 20 per group, respectively). Data are mean ± SEM.

Next, we determined if suramin could reduce renal inflammation in *Pkd1*-miR Tg mice as has been shown in other kidney disease models [33,34,35]. Immunohistochemical staining revealed a significant reduction (53.9%) in the percentage of F4/80 positive area in the kidneys of suramin-treated *Pkd1*-miR Tg mice compared with those treated with vehicle (*p* < 0.01) (Figure 4A). Furthermore, *Pkd1*-miR Tg mice treated with suramin also showed a significant decrease in the renal mRNA expression of *Il1b*, *Il6*, *Nlrp3*, *Emr1* (F4/80), *Tnf*, *Mcp1, P2ry2*, and *P2rx7* compared with vehicle-treated mutant mice (Figure 4B). These results suggest that suramin exerted strong anti-inflammatory effects on the cystic kidneys, which could contribute to the reduced cyst burden.

### 2.5. Effects of Suramin on Renal Fibrosis

**Figure 5:**
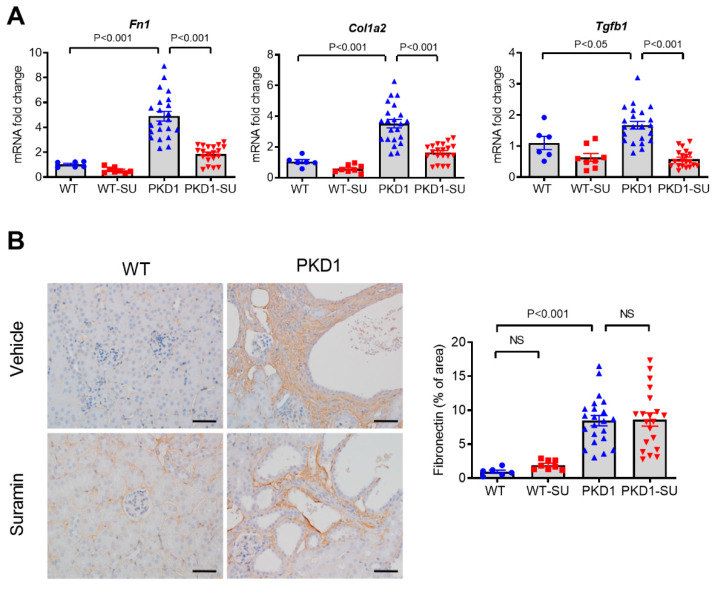
Effect of suramin treatment on renal fibrosis in *Pkd1*-miR Tg mice. (**A**) Comparison of the renal *Fn1, Col1a2,* and *Tgfb1* mRNA expression in different study groups as indicated. The internal control gene used for normalization was rodent 18S ribosomal RNA. (**B**) Immunohistochemistry staining for fibronectin in the kidneys of wild-type and *Pkd1-*miR Tg mice. Quantitative analysis showed increased fibronectin deposition in the cystic kidneys of *Pkd1*-miR Tg mice, but no significant changes were noted after treatment with suramin. Scale bar, 50 µm. Data are mean ± SEM. n = 6, 8, 22, and 20 per group, respectively.

Because a previous study demonstrated that a single dose of suramin suppressed renal fibrosis after acute injury in the mouse UUO model [26], we determined the effect of suramin on renal fibrosis in PKD. We found that the renal mRNA levels of *Fn1, Col1a2,* and *Tgfb1* were significantly lower in the suramin-treated group when compared with the vehicle-treated group (Figure 5A). However, immunohistochemical staining showed no discernible difference in the expression of fibronectin in the kidneys of suramin- or vehicle-treated *Pkd1*-miR Tg mice (Figure 5B). These results indicate that suramin had no obvious antifibrotic effect in the current PKD model despite its strong anti-inflammatory activity.

### 2.6. Suramin Reduces ERK Phosphorylation in Cystic Kidneys

**Figure 6:**
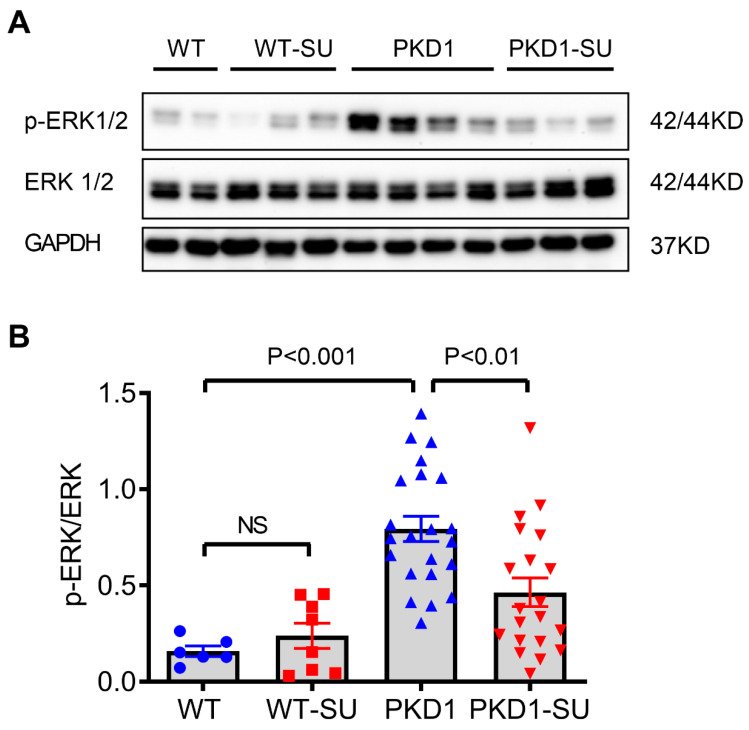
Suramin reduces the phosphorylation of ERK1/2 in *Pkd1-*miR Tg mice. Kidney lysates from *Pkd1-*miR Tg mice and wild-type mice with or without suramin treatment were analyzed by Western blotting analysis. (**A**) Representative immunoblots of phosphorylated ERK1/2, total ERK1/2, and GAPDH. (**B**) The densitometric analysis of p-ERK1/2 normalized to total ERK1/2 protein expression. GAPDH was used for loading control. Data are mean ± SEM (n = 6, 8, 22, and 20 per group respectively).

The activation of the ERK pathway is essential for cAMP-dependent cyst growth in ADPKD [36]. Furthermore, the ERK pathway is one of the downstream effectors of the purinergic receptors [37]. Therefore, we determined whether the increased ERK phosphorylation in cystic kidneys could be suppressed by suramin treatment. Indeed, Western blot analysis showed that the levels of phosphorylation of ERK1/2 in the kidneys of *Pkd1*-miR Tg mice were significantly reduced in the suramin group compared with those in the vehicle group (*p* < 0.01) (Figure 6 and Supplementary Figure S1). These results suggest that suramin could suppress cyst growth through the inhibition of ERK-dependent signaling pathways.

### 2.7. Effect of Suramin on Renal Function and Kidney Injury Markers

**Figure 7:**
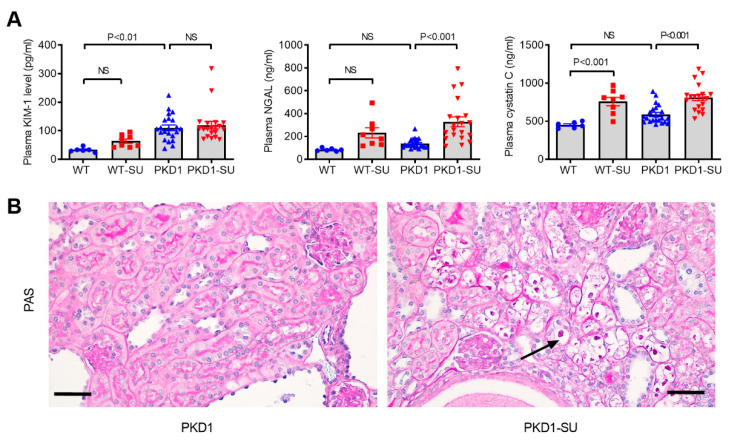
Suramin induces cytoplasmic vacuolation and kidney injuries in non-cystic tubules. (**A**) Plasma levels of kidney injury markers (KIM-1, NGAL, cystatin C) were increased in the suramin-treated *Pkd1-*miR Tg mice compared to the vehicle-treated mutant mice. Data are mean ± SEM (n = 6, 8, 22, and 20 per group respectively). (**B**) Representative periodic acid-Schiff (PAS) staining in the kidney sections from the vehicle- and suramin-treated *Pkd1*-miR Tg mice (n = 3 per group). Arrow indicates the cytoplasmic vacuolation and degeneration of proximal tubules. Scale bar, 50 µm.

Despite the reduction of cyst burden and macrophage infiltration, the elevated BUN levels in *Pkd1*-miR Tg mice were not improved after treatment with suramin. Furthermore, the plasma cystatin-C and Ngal levels in the suramin-treated *Pkd1*-miR Tg mice were significantly higher than those in the vehicle controls by 1.4- and 2.4-fold, respectively (*p*< 0.001) (Figure 7). These unexpected findings suggested that suramin could have caused a nephrotoxic injury. Further histological examination with PAS staining showed multiple vacuolar degenerative changes in the proximal renal tubules in suramin-treated *Pkd1*-miR Tg mice. These results suggest that the accumulation of suramin could induce renal tubular injury and phospholipidosis, which may counteract its beneficial effects on reducing cyst growth and kidney inflammation.

## 3. Discussion

This study determined the effects of suramin, a pan-purinergic receptor antagonist, on the disease progression of PKD in a *Pkd1*-knockdown mouse model. To the best of our knowledge, this is the first in vivo study examining the role of suramin in ADPKD progression. We showed that suramin significantly reduced cyst growth and suppressed renal macrophage infiltration in *Pkd1-*deficient mice. Our results are in line with previous in vitro studies which show an inhibitory effect of suramin on cyst growth in ADPKD [13,38]. A recent bio-chemoinformatic analysis also identified suramin as a candidate drug for ADPKD [39]. However, we found that the administration of suramin also induced degenerative kidney injuries and phospholipidosis in the non-cystic tubules. These results revealed a novel dual role of suramin in modulating PKD progression; it may inhibit the purinergic pathway and cyst growth but can also induce phospholipidosis and tubular cell injuries.

Suramin has demonstrated potent anti-inflammatory and antifibrotic properties in various kidney disease models but has not been tested in ADPKD [26,33,35,40]. Indeed, we found that suramin markedly inhibited inflammatory cytokines and macrophage infiltration in the cystic kidneys, which was associated with reduced cyst growth. These findings support the emerging concept that the inhibition of macrophage infiltration surrounding kidney cysts could modify the cyst microenvironment and reduce the proliferation of cyst-lining epithelial cells [41,42,43]. However, we did not observe a significant reduction of renal fibrosis after treatment with suramin. The discrepancy could be attributed to different disease-specific mechanisms of renal injury and fibrosis in these animal models and the time frame of suramin administration (i.e., single-dose vs repeated doses). The defective lysosomal degradation of extracellular matrix proteins in ADPKD could be further aggravated by suramin-induced lysosomal damage, which may counteract its potential anti-fibrotic effect [44,45,46].

One important finding of the current study is the unexpected tubular injury following suramin treatment. These findings are consistent with earlier clinical trials, which have demonstrated that suramin can cause neurotoxicity and nephrotoxicity in patients with prostate cancer [47]. Acute non-oliguric kidney injury has been reported in a patient with prostate cancer following suramin treatment [48]. Suramin is highly protein-bound and has a long elimination half-life (36 to 54 days) from urinary excretion [49]. In animal experiments, suramin has been shown to induce glycosaminoglycan and sphingolipid accumulation in the renal tubular lysosome [48,50]. A recent study showed that suramin may induce phospholipidosis via the inhibition of lysosomal phospholipase A2 [51]. The dose of suramin used in the current study is still within the non-cytotoxic concentration range according to previous studies in mouse models of cardiac fibrosis and lung injury [28,29,30]. Although further studies using other animal models for PKD and different dosing schedules are needed to confirm our results, our findings raised an important safety concern about using suramin in the setting of PKD due to drug-induced kidney injury and lysosomal damage. The results also highlight the importance to evaluate the effects of candidate compounds on the non-cystic tubules and glomeruli, which are responsible for maintaining normal kidney function.

## 4. Materials and Methods

**Animals:***Pkd1* miRNA transgenic (referred to as *Pkd1*-miR Tg) mice that present stable and heritable *Pkd1* knockdown (~70%) were used to determine the effects of suramin [27]. This *Pkd1*-deficient model is characterized by a moderately progressive disease course that mimics the gradual cyst development in ADPKD [52]. Wild-type littermates (C57BL/6) were used as controls. Mice were housed under climate-controlled conditions with a 12-h light–dark cycle and free access to drinking water and standard rodent chow. The study was approved by the Animal Care and Use Committee of Chang Gung Memorial Hospital and conformed to the National Research Council’s Guide for the Care and Use of Laboratory Animals.

**Experimental design:***Pkd1-*miR Tg mice and wild-type littermates of both genders were allocated to experimental and control groups randomly. The experimental groups received twice-weekly i.p. injections of suramin (60 mg/kg, Tocris Bioscience, Bristol, UK) from P35 for eight weeks according to the effective and non-cytotoxic dose found in mouse models of heart and lung fibrosis [28,29,30]. The control groups received the same volume of vehicle (normal saline) by i.p. injections. Mice were sacrificed on P90 and blood samples were obtained by cardiac puncture under anesthesia. Both kidneys were harvested and weighed immediately. Tissue samples were snap-frozen in liquid nitrogen and stored at −80 ℃ for further experiments.

**Kidney function tests:** Blood urea nitrogen (BUN) levels were measured using VITROS DT60 II Chemistry System (Ortho Clinical Diagnostics, Rochester, NY, USA). The plasma levels of cystatin C, neutrophil gelatinase-associated lipocalin (NGAL) and the kidney injury molecule-1 (KIM-1) were measured using mouse ELISA kits (R&D Systems, Minneapolis, MN, USA) according to the manufacturer’s instructions [53].

**Histomorphometric analysis:** Kidney samples were fixed in 10% neutral-buffered formalin overnight and transferred to 75% ethanol before being embedded in paraffin for sectioning. Hematoxylin and eosin (H&E) staining and periodic acid-Schiff (PAS) staining were performed on transverse kidney sections according to the standard methods. The cystic index was calculated by dividing the area of the cystic lumen by the total kidney area using MetaMorph software (Universal Imaging, West Chester, PA, USA) [54]. All analyses were performed on coded slides in a single-blinded manner.

**Immunohistochemistry:** Kidney sections (4-μm thick) were dewaxed, rehydrated, and boiled in citrate buffer (pH 6.0) for antigen retrieval. The slides were blocked with Dako Antibody Diluent (Cat. No. S3022; Dako Agilent Technologies, Santa Clara, CA, USA). Renal macrophages were recognized using a rat anti-mouse F4/80 antibody (1:100, MCA497R; Serotec, Oxford, UK) overnight at 4℃, followed by a biotin-conjugated goat anti-rat light chain antibody (1:1000, AP202B; Sigma-Aldrich, Saint Louis, MO, USA) and streptavidin–horseradish peroxidase (HRP) label (HP604H, Biocare Medical, Concord, CA, USA) 30 min at room temperature. Fibronectin and P2X7 receptor immunostaining were performed using a rabbit anti-fibronectin antibody (1:250, ab2413; Abcam, Cambridge, UK) and a rabbit polyclonal anti-P2X7 receptor ATTO 550 antibody (1: 50, APR 004 AO; Alomone Laboratories, Jerusalem, Israel) overnight at 4℃, respectively. The signals were detected by using Rabbit-on-Rodent HRP-Polymer (RMR622H; Biocare Medical, Concord, CA, USA) for 30 min at room temperature. A positive reaction was visualized using 3,3′-diaminobenzidine chromogens (K3468; DAKO, Glostrup, Denmark). The slides were counterstained with hematoxylin (Muto Pure Chemicals, Tokyo, Japan). The percentage of F4/80 or fibronectin was calculated using five consecutively selected fields of the renal cortex and medulla at 100× magnification in the sections.

To determine the degree of cell proliferation, mice received an i.p. injection of 5-Bromo-2′-deoxyuridine (BrdU, 0.3 mg/kg) (Sigma-Aldrich, St. Louis, MO, USA) four hours before sacrifice. The kidney sections were treated with 2 N HCl for antigen retrieval. BrdU-positive cells were detected by a rabbit monoclonal anti-BrdU antibody (1:1000, ab6326, Abcam, Cambridge, UK). The proliferative index was determined by counting the number of BrdU-positive epithelial cells in sequentially selected fields in each renal section at 200× magnification.

**Quantitative polymerase chain reaction:** Total RNA was extracted from snap-frozen kidney tissues using TRIzol reagent (Invitrogen, Carlsbad, CA, USA) according to the manufacturer’s protocol. cDNA was obtained from the total RNA using a Transcriptor First Strand synthesis kit (Roche, Indianapolis, IN). A quantitative real-time polymerase chain reaction (qRT-PCR) was performed using an ABI ViiA7 sequence detection system (Applied Biosystems, Foster City, CA, USA) with TaqMan assays or SYBR Green primers as listed (Supplementary Table S2). The reactions for each sample were performed in duplicate. The relative mRNA expression levels were calculated using the 2-ddCt method. Rodent 18S ribosomal RNA was used as an endogenous control.

**Western blotting:** Proteins were isolated from homogenized frozen kidney specimens. Equal amounts of total protein were separated by sodium dodecyl sulfate-polyacrylamide gel electrophoresis and then transferred onto a polyvinylidene fluoride membrane (Millipore, Bedford, MA, USA). After blocking in 5% fat-free milk, the membrane was incubated individually with the primary antibodies, including rabbit polyclonal anti-phospho-extracellular signal-regulated kinase (ERK1/2) (Thr202/Tyr204) (1:1000, #9101), rabbit monoclonal anti-ERK1/2 (137F5) (1:1000, #4695; Cell Signaling Technology, Danvers, MA, USA), and rabbit monoclonal anti-GAPDH (EPR16891) (1:10000, ab181602; Abcam, Cambridge, UK), at 4 °C overnight. The membranes were further incubated with HRP-conjugated secondary antibodies at room temperature and the signal was detected through the enhanced chemiluminescence method.

**Statistical analysis:** Values are expressed as mean ± SEM. Between-group comparisons were performed using one-way analysis of variance (ANOVA) followed by the Dunnett’s multiple comparison test. *p* values < 0.05 were considered statistically significant. All analyses were performed using GraphPad Prism 9 (GraphPad, La Jolla, CA, USA).

**Conclusions:** In summary, the study shows that suramin reduced cyst growth and suppressed renal inflammation in a *Pkd1*-deficient mouse model of ADPKD. However, suramin also induced kidney injuries in non-cystic tubules and overall did not slow the decline of renal function. Our findings do not support a repurposed role of suramin in the treatment of ADPKD progression. Further studies using other purinergic receptor antagonists with less nephrotoxicity may help to elucidate the therapeutic potential of targeting purinergic signaling in ADPKD.

## Data Availability

Not applicable.

## Supplementary Materials

The supporting information can be downloaded at: https://www.mdpi.com/article/10.3390/ijms23158499/s1.
