# Supplementary material for: Effects of Suramin on Polycystic Kidney Disease in a Mouse Model of Polycystin-1 Deficiency
Source: Int J Mol Sci. 2022 Jul 31;23(15):8499. doi: 10.3390/ijms23158499 (PMC9369130; doi:10.3390/ijms23158499)
Supplement: Supplementary file 1 [file ijms-23-08499-s001.zip › ijms-1833993-supplementary.pdf]

Supplementary Table S1. Effects of suramin on body weight and kidney weight of wild-type and *Pkd1*-miR Tg mice

	**WT+Vehicle (n=6)**	**WT+Suramin (n=8)**	***Pkd1*+Vehicle (n=22)**	***Pkd1*+Suramin (n=20)**
Gender, F/M	3/3	4/4	10/12	11/9
Body weight (g), M	26.9± 2.0	23.0 ± 0.8*	25.1 ±0.5	23.3 ± 0.5*
Body weight (g), F	22.4 ± 0.2	20.9 ± 0.9	21.5 ± 0.3	18.6 ± 0.6**,$^{###}$
Kidney weight (g), M	0.35 ± 0.03	0.30± 0.02	0.61 ± 0.02***	0.56± 0.03***
Kidney weight (g), F	0.26 ± 0.00	0.26 ± 0.01	0.45± 0.03**	0.36 ± 0.02$^{\#}$
Kidney-to-body weight ratio (%)	1.23 ± 0.03	1.29± 0.06	2.28± 0.09***	2.16 ± 0.09***

Values are mean ± SEM.$^{*}P < 0.05$, $^{**}P < 0.01$, and $^{***}P < 0.001$ versus wild-type mice (WT) treated with vehicle; $^{\#}P < 0.05$, $^{###}$ $P < 0.001$ versus *Pkd1*-miR Tg mice treated with vehicle.

Supplementary Table S2. TaqMan probe ID and primer sequences

Gene	***Taqman probe or primer sequences (5' to 3')***
Col1a2	F: AAGGGGTCTTCCTGGTGAAT<br>R: GGGGTACCACGTTCTCCTC
Fn1	F: TGTGACCAGCAACACGGTG<br>R: ACAACAGGAGAGTAGGGCGC
Tgfb1	F: TGCGTCTGCTGAGGCTCAA<br>R: TTGCTGAGGTATCGCCAGGA
***Emr1* (F4/80)**	Mm00802529_m1
Il1b	Mm00434228_m1
Il6	Mm01210733_m1
Mcp1	Mm00441242_m1
Nlrp3	Mm00840904_m1
P2rx7	Mm00440578_m1
P2ry2	Mm02619978_s1
Rn18S	Mm03928990_g1
Tnf	Mm99999068_m1

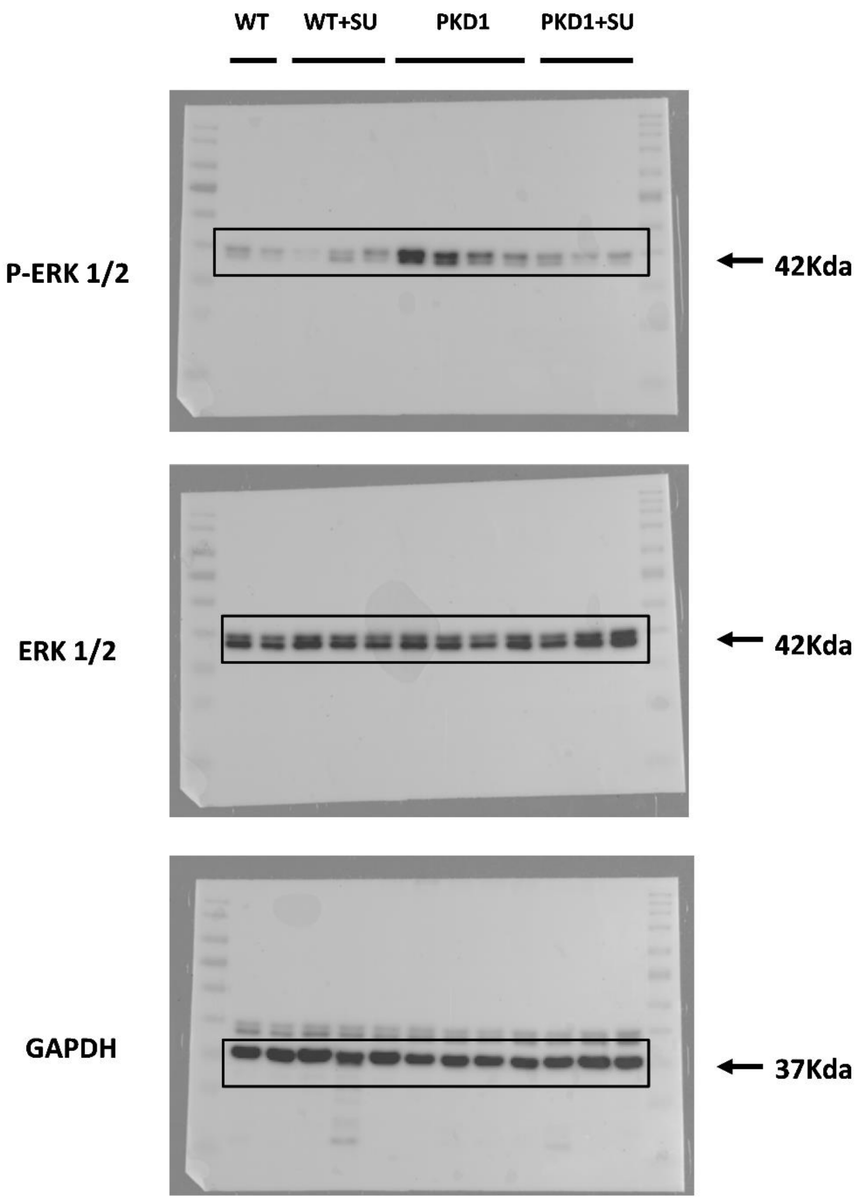

Supplementary Figure S1. Uncropped Western blot images of Figure 6.
